# The Open Source Seed Licence: A novel approach to safeguarding access to plant germplasm

**DOI:** 10.1371/journal.pbio.3000023

**Published:** 2018-10-23

**Authors:** Johannes Kotschi, Bernd Horneburg

**Affiliations:** 1 Agrecol, Guggenhausen, Germany; 2 Division of Plant Breeding, CiBreed, University of Göttingen, Göttingen, Germany

## Abstract

The laws to secure intellectual property rights on plant germplasm have been strongly developed in parallel to the ongoing seed market consolidation. Germplasm as a commons, i.e., a natural resource accessible to all members of a society, receives almost no legal protection. On the other hand, the use of germplasm and released cultivars in breeding is increasingly restricted by intellectual property rights. In this study, approaches to open source plant germplasm are discussed, and the Open Source Seed (OSS) Licence is introduced and analysed. The OSS Licence was developed by an interdisciplinary working group of plant breeders, agricultural scientists, lawyers, and commons experts in Europe. The aim is to protect germplasm as a commons, support the free exchange of germplasm, stimulate plant breeding, reduce costs, and accelerate innovation. The OSS Licence is a legal tool and novel approach that extends its reach on derivatives of licenced germplasm. It is compatible with current seed laws. Effects on the access to plant germplasm, on breeding for diverse pedoclimatic environments, socioeconomic systems, and on biodiversity as a whole can first show after a few breeding cycles. The impact of open source germplasm on these aspects needs to be monitored carefully.

## Abstract 2

This Community Page article presents the Open-Source Seed Licence, developed by an interdisciplinary working group of plant breeders, agricultural scientists, lawyers and commons experts in Europe. The aim is to protect germplasm as a commons, support the free exchange of germplasm, stimulate plant breeding, reduce costs and accelerate innovation.

## Introduction

For millennia, crop seed has been a common good [1], and all over the world, crops have been cultivated, enhanced, and bred by farmers, a practice that resulted in a rich inter- and intraspecific diversity of crops. The emergence of agricultural sciences at the end of the 19th century marks an historical change to separating plant breeding from plant production [2]. Simultaneously, scientific plant breeding began contributing decisively to agricultural intensification. In the 1940s, combining high-yielding crops with extensive use of mineral fertilizers and chemical plant protection became the main strategy for food security [3,4]. High-yielding cultivars were developed using breeding techniques such as hybrid breeding, facilitated through cytoplasmic male sterility systems that prevent the use of farm-saved seed [5]. Uniform cropping systems with only a few crops and cultivars spread over large areas. At later stages, intellectual property rights such as plant variety protection and utility patents were implemented [6,7]. Increasing market consolidation led to a few transnational corporations dominating the global seed market [8,9]. Consequently, the number of breeding programs was greatly reduced, and decreasing competition between companies has restricted innovation [10,11,12]. There is also a significant loss of cultivars caused by consolidation, as companies reduce portfolio size after mergers. Economies of scale act in favor of large-scale propagation of single cultivars and against breeding for diverse pedoclimatic conditions. The access to plant genetic material is increasingly constrained for farmers, plant breeders in public institutions [13], small breeding companies, and public domain initiatives. Thus, after genetic bottlenecks significantly reduced diversity from early domesticates to modern cultivars, [14] the next bottleneck has been created by intellectual property rights and market consolidation.

The loss of agrobiodiversity is critical. Plant genetic diversity is essential for food security as a means of adaptation to variable weather conditions, as it enhances social and ecological resilience against extreme events [15,16,17]. A rich diversity of crops and cultivars is required for the world’s innumerable agroecological sites and corresponding farming systems. Cultivars are needed that not only satisfy the needs of large-scale farming but also give smallholders—by far the majority of farmers in the world—a livelihood that allows them to contribute adequately to global food supply in quantity and quality [3,18]. The demand cannot be met by the private seed sector alone [11].

While public sector plant breeding has undergone a steady decline in Europe [19], new stakeholders have evolved. During the past 40 years, public domain initiatives, mainly in the context of organic agriculture, have been established [20]. In many countries of the Global South, participatory plant breeding with smallholders has been established successfully [21,22,23]. Also, in large international institutions of the Consultative Group on International Agricultural Research (CGIAR), open access systems have been effectively applied for crop improvement [24,25]. Most of the organic plant-breeding programs in Europe are organised by associations, foundations or informal networks and operate on a nonprofit basis. Most new cultivars are released “open access,” i.e., without intellectual property rights, unconditionally and to everybody. This allows users to privatize subsequent derivatives or reproductions of that plant germplasm. In other words, commons are created but not protected.

Although the International Treaty on Plant Genetic Resources for Food and Agriculture [26] recognizes so-called “farmers’ rights” to use farm-saved seed, much tension has developed between farmers’ rights and intellectual property rights of the private sector, in particular plant variety protection and patenting, in international governance [27,28]. Access to plant germplasm for the nonprivate seed sector is not sufficiently granted.

As a new tool, the Open Source Seed Licence for plant germplasm (new cultivars, breeding lines, and populations) was developed by an interdisciplinary working group of the nongovernmental organization (NGO) Association for AgriCulture and Ecology (Agrecol). The primary target area is Europe. We will describe and discuss i) approaches to create open source plant germplasm, ii) the Open Source Seed Licence and the steps needed for its implementation, iii) the analysis of existing seed laws for potential conflicts with open source plant germplasm, and iv) potential effects of the Open Source Seed Licence on breeding for diverse pedoclimatic and social environments and on access to plant germplasm.

## Approaches to create open source plant germplasm

The term “open source” is derived from computer science, for which it refers to free access to computer source codes. The open source concept is a development of the free software principles first articulated in the GNU General Public Licence [29] by Richard Stallman, already declared in the GNU Manifesto [30]. The aim was to allow developers and users of computers to investigate how software works and be able to modify and share their work freely with others. Several authors have suggested the analogy of plant breeding with software development [31,32,33,34]. The open source rules for plant germplasm imply that anybody may use plant germplasm for production, breeding, multiplication, and distribution. At the same time, any user is obliged to impose the same rights on future owners of the plant germplasm. Any limitation beyond this is prohibited. This clause is also called “copyleft.” Thus, open source is clearly distinguished from open access, which implies unlimited access. Essentially, open source safeguards access to a common good and is therefore creating a protected commons preventing privatization [35]. During the past six years, approaches to create open source plant germplasm have evolved in the United States [36], in Germany [37], in India [38], and in East Africa [39].

The Open Source Seed Initiative (OSSI) was founded 2012 in the US by a group of academic and nonacademic plant breeders, farmers, nonprofit agencies, seed advocates, and policymakers as the first organization for the development of open source seeds [36]. Inspired by Michaels [31], who had already presented the idea of a General Public Licence for plant germplasm in 1999, OSSI developed a licence. But the licence proved too cumbersome for practical use, and subsequently, the OSSI Pledge was developed as a social mechanism that requests an ethical commitment from seed users to comply with the open source rules [13]. The pledge is used by dozens of plant breeders and seed companies internationally to create an “ethical sharing and distribution mechanism for plant breeding” [36].

The Dutch NGO Hivos started the “Open Source Seed Systems Programme” [39]. It involves cooperation with partners in East Africa and India. It is Hivos’ strategy to work through multistakeholder initiatives to search for alternatives, build viable business models for open source seeds, and seek support through lobbying and advocacy. As yet, no legal and/or ethical commitment is being used.

The Apna Beej Network in India also practices an “Open Source Seed System”. It works with a “material and knowledge transfer agreement” that allows members of the network to use their plant germplasm freely within the network [38].

The German NGO Agrecol started work on open source plant germplasm systems in 2012 [37]. An interdisciplinary working group of agricultural scientists, plant breeders, and lawyers compared ethical approaches using a pledge with legal approaches using a licence. A pledge seemed to meet acceptance by potential users more easily than a licence. On the other hand, it is less binding than a licence, as it might be withdrawn or disregarded. In addition, a licence meets favorable conditions for legal enforcement in the European Union (EU): according to the Nagoya Protocol and unlike in the US, the mandatory documentation of germplasm that is used for new cultivars facilitates tracing licence infringements. Consequently, the Open Source Seed (OSS) Licence was developed and published in 2016 [40]. This in turn inspired apiculturists from Apimondia, the World Beekeepers Association, to use an open source licence for honey bees [41], and the first animal genetic open source licence was developed.

## The Open Source Seed Licence

The OSS Licence grants users many freedoms and demands little: the licensee is obliged to grant the same rights he or she has enjoyed to future owners of the plant germplasm. This obligation is “viral” and includes all derivatives of the germplasm. With the first licensing, a chain of contracts is started, which, in principle, is endless. Licensees become licensors, who pass on the germplasm with the same licence.

Legally, the OSS Licence is a “sui generis contract” and falls under the General Business Terms and Conditions of German Civil Law [42], a prewritten contract for general, multiple, and unilateral use by a single party and not individually negotiated. The licence is a material transfer agreement for seeds and vegetative parts of plants. It confers user rights together with the material object and its genetic information. When the material is transferred, a contract is established that ensures the rights and duties associated with the material in question as well as all future derivatives and reproductions of that plant germplasm. The OSSI pledge also intends to do this, but unlike a pledge, the licence can be used to legally protect newly developed cultivars, breeding lines, populations, and other germplasm. Thus, the open source character of germplasm can be legally enforced. This is a novelty.

To transfer germplasm under the OSS Licence, the licence conditions must, unambiguously, be disclosed. This means that any transfer is valid if the licensee is able to become aware of the terms and conditions of the licence. For traders, this means that an abridged version of the OSS Licence must be printed on the wrapping of small bags with reference to the online full-text version. For larger quantities, the licensors must ensure that a copy of the licence accompanies the material being transferred, and they must explicitly inform the recipient (the licensee). Licence infringements can be examined through a combination of measures. The most important one is to analyze the breeding process that the new cultivar has undergone. According to the Nagoya Protocol [43], a detailed documentation for each breeding process is mandatory. The user of plant genetic resources must record the time and place of access and, where appropriate, also examine “the presence or absence of rights and obligations relating to access and benefit sharing” [44]. Thus, the Nagoya Protocol is a strong lever to enforce the OSS Licence. Additionally, phenotypic and genotypic data can be used. Monitoring of licence infringements is considered the responsibility of all users to ensure that open source remains open source. OpenSourceSeeds aims to initiate a social process to this effect because licence infringements could have negative repercussions for everyone.

The OSS Licence was implemented on April 26, 2017, when Agrecol launched the service provider OpenSourceSeeds [45] and presented the first OSS-licensed cultivars in Berlin, Germany. OpenSourceSeeds, under the legal entity of Agrecol, licenses newly developed cultivars on behalf of breeders. It documents OSS licensed germplasm in a database and stores samples of plant germplasm. In addition, the development of value chains with OSS-licensed cultivars is supported.

OpenSourceSeeds started with two cultivars, a vegetable and a field crop. The tomato Sunviva was bred by the network of the Organic Outdoor Tomato Project launched in 2003 by the University of Göttingen. The project is based on participation and the free exchange of tomato genotypes and related knowledge [46]. The project aims to improve plant-breeding methodology [47]. Culinaris, a seed company that exclusively sells seeds free of intellectual property rights, is in charge of legal issues and maintenance breeding. The spring wheat Convento C was developed by Forschung & Züchtung Dottenfelder Hof in Bad Vilbel, Germany. Meanwhile, seven cultivars of different crops including maize have been licensed.

## Discussion and outlook

The laws on securing intellectual property rights on plant germplasm have been strongly developed, whereas plant germplasm as a commons receives almost no legal protection. Thus, the possibility to license new cultivars as open source is new and unfamiliar. The release of the first OSS-licensed cultivars will stimulate further discussions and generate valuable experiences on the feasibility, acceptance, and impact of open source germplasm among plant breeders, seed producers, growers, plant genetic resources experts, and society as a whole.

Being a legal tool, the question may arise whether the OSS Licence is in conflict with existing seed laws on seed marketing and plant variety protection. In the EU, national laws like the German Seed Marketing Act are based on EU regulations. The German Seed Marketing Act regulates the release of most agricultural and horticultural crops. The act is not in conflict with OSS licensing, but germplasm of the relevant crops can only be legally released when the cultivars are registered in the Plant Variety Database of the European Commission. The European Plant Variety Protection Act is not in conflict with open source germplasm because it only applies for cultivars under plant variety protection.

Another issue to be addressed is the funding of a future plant breeding that needs to supply cultivars adapted to very diverse pedoclimatic conditions as a service for society. Financing models are to be further developed that are not based on royalties on intellectual property rights. In many developing countries, plant breeding does not follow a business model based on royalties, and even in industrial countries, there are private breeding companies that do not rely on intellectual property rights. Presently, many organic plant breeders in Europe finance their breeding work partly through “cultivar development contributions” that are negotiated between breeders, seed producers, and farmers. Some have, in cooperation with the food trade, developed a levy on food items, and most of them raise funds from government programs and foundations [48]. The funds for commons-based plant breeding are still small but continuously increasing.

Incentives for the application of the OSS Licence may be manifold. At present and after the first year of introduction, a significant demand from consumers can be observed. Interviews indicate that the public appreciates the possibility to contribute to an alternative to the dominant system of privatization and market concentration in the seed sector. Exemplarily, this can be observed with the OSS-licensed tomato cultivar Sunviva. Due to the growing demand in horticulture, more and more traders are offering Sunviva seed and its seedlings. Consumers could, therefore, create a significant pull effect. Other incentives may be identified in the following years. We expect that the increasing shortage of freely available breeding material will create a strong incentive for small and medium plant breeders to license their new cultivars open source and to contribute to a commons-based pool of plant germplasm.

Expected impacts of the licence will have to be assessed in the following areas. Open source systems aim to allow plant breeders to operate and cooperate freely [36]. It is assumed that a free exchange of germplasm stimulates plant breeding, reduces costs for breeding, and accelerates innovation. This might counteract the current trend of consolidation in the seed market and increase number and diversity of breeding programs. New plant-breeding methods like participatory plant breeding might be facilitated, which can offer cheap and fast methods of cultivar development [22]. All of these aspects can be essential to sustain diversity of plant germplasm.

We expect insights into the acceptance of the OSS Licence in the next few years because breeders and researchers can decide immediately how they release new genotypes. Monitoring the effects of licensing open source plant germplasm on other areas of major importance, however, will last much longer. Effects on the access of various stakeholders to plant germplasm, on breeding programs for diverse pedoclimatic and social environments and on biodiversity as a whole can first show after a few breeding cycles. A network meeting to evaluate first experiences with the OSS Licence will take place in 2019. We expect specific effects for different crop types (minor or major crop, field crop, vegetable, fruit trees), types of cultivars (pure line, multiline, population), and breeding systems (autogamous, allogamous, clones). The impact of open source germplasm on the areas discussed above will have to be monitored carefully.

## Abbreviations

Agrecol: Association for AgriCulture and Ecology
CGIAR: Consultative Group on International Agricultural Research
EU: European Union
NGO: nongovernmental organization
OSSI: Open Source Seed Initiative
OSS: Open Source Seed
